# Development of a multiplex isothermal amplification molecular diagnosis method for on-site diagnosis of influenza

**DOI:** 10.1371/journal.pone.0238615

**Published:** 2020-09-11

**Authors:** Woong Sik Jang, Da Hye Lim, Jeonghun Nam, Do-CiC Mihn, Haan Woo Sung, Chae Seung Lim, Jeeyong Kim

**Affiliations:** 1 Department of Laboratory Medicine, Korea University Guro Hospital, Seoul, Republic of Korea; 2 Department of Diagnostic Immunology, Seegene Medical Foundation, Seoul, Republic of Korea; 3 Department of Veterinary Microbiology, College of Veterinary Medicine, Kangwon National University, Chuncheon, Republic of Korea; University of Georgia, UNITED STATES

## Abstract

Influenza, which is an acute respiratory disease caused by the influenza virus, represents a worldwide public health and economic problem owing to the significant morbidity and mortality caused by its seasonal epidemics and pandemics. Sensitive and convenient methodologies for the detection of influenza viruses are important for clinical care and infection control as well as epidemiological investigations. Here, we developed a multiplex reverse transcription loop-mediated isothermal amplification (RT-LAMP) with quencher/fluorescence oligonucleotides connected by a 5′ backward loop (LF or LB) primer for the detection of two subtypes of influenza viruses: Influenza A (A/H1 and A/H3) and influenza B. The detection limits of the multiplex RT-LAMP assay were 10^3^ copies and 10^2^ copies of RNA for influenza A and influenza B, respectively. The sensitivities of the multiplex influenza A/B/IC RT-LAMP assay were 94.62% and 97.50% for influenza A and influenza B clinical samples, respectively. The specificities of the multiplex influenza A/B/IC RT-LAMP assay were 100% for influenza A, influenza B, and healthy clinical samples. In addition, the multiplex influenza A/B/IC RT-LAMP assay had no cross-reactivity with other respiratory viruses.

## Introduction

Influenza, which is caused by the influenza virus, is a significant cause of morbidity and mortality that has major social and economic impacts throughout the world [1, 2]. Iuliano *et al*. reported that 291,243–645,832 seasonal influenza-associated respiratory deaths occur annually (4·0–8·8 per 100,000 individuals) [3]. Influenza viruses belong to the Orthomyxoviridae family and have a single-stranded segmented RNA genome consisting of 7–8 segments encoding 10–11 proteins [4]. The influenza viruses are classified into types A, B, C, and D on the basis of their core proteins [5]. Among the four influenza types, influenza A viruses cause most of the global flu epidemics, influenza B viruses cause smaller localized outbreaks, influenza C viruses generally cause mild illness, and influenza D viruses are not known to cause illness in people [6–8]. Thus, influenza A and B viruses comprise the major respiratory pathogens in humans that cause seasonal flu epidemics [9, 10].

The three kinds of therapeutic treatments for influenza A and B viruses include M2 inhibitors (amantadine and rimantadine), neuraminidase (NA) inhibitors (zanamivir and oseltamivir) and cap-dependent endonuclease (CEN) inhibitor (baloxavir) [11–14]. NA inhibitors and CEN inhibitors have both inhibitory effects on influenza A and influenza B, but M2 inhibitors act specifically on influenza A [15, 16]. Since antiviral drugs can reduce the severity of illness when administered within 48 h of the first symptoms, distinguishing between influenza A and B is critical for choosing the appropriate antiviral drug [17, 18].

Currently, diagnosis methods for influenza viruses include the antigen antibody test, the hemagglutination inhibition test, enzyme immunoassay, microscopic diagnosis, and molecular diagnosis [19–21]. However, serological tests have low sensitivity and specificity in comparison with PCR and PCR-based assays requiring expensive instruments, specialized technicians, and complicated procedures; thus, all these methods are unsuitable for rapid diagnosis in field situations [22, 23]. In 2000, loop-mediated isothermal amplification (LAMP) was developed to amplify genes at constant temperatures. The LAMP assay is a rapid, highly sensitive isothermal nucleic acid amplification through chain substitution reaction. LAMP amplifies the target gene at 60–65°C with six primers, including four primers selected by combining six parts of a target DNA strand and two additional loop primers. Bst DNA polymerase, which is a strand displacement DNA polymerase, is used in the LAMP assay to enable loop structure formation of the inner primers, producing LAMP’s unique rapid self-priming amplification [24, 25]. The LAMP assay has been widely applied for the detection of various pathogens [26–28]. In particular, the reverse transcription LAMP (RT-LAMP) assay for RNA viruses is widely used for point-of-care testing because it does not require the standard RT reaction time [29].

For multiple detection using the LAMP assay, an assimilating probe consisting of the fluorescently tagged probe and its complementary sequence probe tagged with quencher was developed. This probe works by separating fluorescently-tagged oligonucleotides from the quencher-tagged probe. As a result, fluorescence is observed in real-time and measured from the fluorescently-tagged probe that has been incorporated into RT-LAMP products [30, 31].

In this study, we present a rapid multiplex RT-LAMP diagnosis method for influenza A, influenza B, and an internal control using a newly designed target-specific assimilating probe and fluorescently-tagged strand displaceable probes. This multiplex influenza A/B/IC RT-LAMP assay had high sensitivities for influenza A and influenza B without interference from one another. Moreover, the assay showed no cross-reactivity against other respiratory and hemorrhagic fever viruses.

## Materials and methods

### Clinical samples and RNA extraction

Nasopharyngeal (NP) swabs were collected from patients presenting flu-like symptoms at Korea University Guro Hospital from January 2018 to December 2018. A total of 314 NP specimens were used in this study, including 100 negative and 214 positive specimens of the following viruses: 11 influenza A/H1, 82 influenza A/H3, 80 influenza B, 4 RSV A, 4 RSV B, 4 adenovirus, 4 parainfluenzavirus (PIV1-4), 9 coronavirus (OC43, NL63, and 229E), 4 human bocavirus (HBoV), 4 human enterovirus (HEV), 4 human rhinovirus (HRV), and 4 metapneumovirus (MPV). All virus specimens were confirmed by polymerase chain reaction (PCR) using an Anyplex II RV16 Detection Kit (Seegene, Inc., Seoul, South Korea). In addition, H5, H7 and H9 subtypes of avian influenza viruses were provided by Department of Veterinary Microbiology, College of Veterinary Medicine, Kangwon National University School of Medicine, Korea. RNA extraction was performed with a QIAamp Viral RNA Mini Kit (Qiagen, Hilden, Germany) according to the manufacturer's manual. RNA was extracted from 140 μL of samples and stored at -50°C. All LAMP assays were performed blindly with the operator unaware of any previous test results. This study was approved by the Medical Ethics Committee of Korea University Guro Hospital (2019GR0055).

### Primer design

Influenza A and B LAMP primer sets were designed within conserved regions of segment 7 of influenza A and the nucleoprotein gene of influenza B. For internal control, actin beta LAMP primer set was newly designed within of conserved regions of human actin beta mRNA (NM_001101.5:c.287-c.498), which is commonly used as internal control [32]. All LAMP primers, including two outer primers (forward primer F3 and backward primer B3), two inner primers (forward inner primer FIP and backward inner primer BIP), and two loop primers (forward loop primer LF and backward loop primer LB), were designed using Primer Explorer software (Version 4; Eiken Chemical Co., Tokyo, Japan). For the multiplex LAMP assay, we designed the fluorophore probe oligomer (32 mer) at the 5′ LF or LB primer and the quencher oligonucleotide (30 mer), which is the complementary sequence of the fluorophore probe oligomer, using Random DNA Sequence Generator (https://faculty.ucr.edu/~mmaduro/random.htm). All primers were assessed for specificity before use in the LAMP assays via a BLAST search of sequences in GenBank (National Center for Biotechnology Information [NCBI], Bethesda, MD). All LAMP primers and probes were synthesized by Macrogen, Inc. (Seoul, South Korea; Table 1).

**Table 1:** Loop-mediated isothermal amplification (LAMP) and Reverse Transcription Polymerase Chain Reaction (RT-PCR) primer sets used in this study.

Target	Name	Sequence (5`-3`)	Length (mer)
Influenza A (segment 7 gene)	INFA F3	GAC TTG AAG ATG TCT TTG C	17
	INFA B3	TRT TAT TTG GGT CTC CAT T	19
	INFA FIP	TTA GTC AGA GGT GAC ARR ATT GCA GAT CTT GAG GCT CTC	39
	INFA BIP	TTG TKT TCA CGC TCA CCG TGT TTG GAC AAA GCG TCT ACG	39
	INFA BLP	CMA GTG AGC GAG GAC TG	16
	INFA FLP	GTC TTG TCT TTA GCC A	17
	INFA FLP probe	[FAM]-CGG GCC CGT ACA AAG GGA ACA CCC ACA CTC CGG TCT TGT CTT TAG CCA	48
Influenza B (NP gene)	INFB F3	GAG CTG CCT ATG AAG ACC	18
	INFB B3	CGT CTC CAC CTA CTT CGT	18
	INFB FIP	GAA CAT GGA AAC CCT TGC ATT TTA AGT TTT GTC TGC ATT AAC AGG C	46
	INFB BIP	GAA CAG RTR GAA GGA ATG GGR GCG ATC TGG TCA TTG GAG CC	41
	INFB FLP	TGC TGA TCT AGG CTT GAA TTC TGT	23
	INFB BLP	AGC TCT GAT GTC CAT CAA GCT CC	24
	INFB FLP probe	[Texas red]-CGG GCC CGT ACA AAG GGA ACA CCC ACA CTC CGA GCT CTG ATG TCC ATC AAG CTC C	55
Human (actin gene)	IC F3	AGT ACC CCA TCG AGC ACG	18
	IC B3	AGC CTG GAT AGC AAC GTA CA	20
	IC FIP	GAG CCA CAC GCA GCT CAT TGT ATC ACC AAC TGG GAC GAC A	40
	IC BIP	CTG AAC CCC AAG GCC AAC CGG CTG GGG TGT TGA AGG TC	38
	IC FLP	TGT GGT GCC AGA TTT TCT CCA	21
	IC BLP	CGA GAA GAT GAC CCA GAT CAT GT	23
	IC BLP probe	[HEX]-CGG GCC CGT ACA AAG GGA ACA CCC ACA CTC CGC GAG AAG ATG ACC CAG ATC ATG T	55
Quencher probe		GAG TGT GGG TGT TCC CTT TGT ACG GGC CCG-BHQ1	30
Influenza A (M gene)	RT-PCR INFA-1	CTTCTAACCGAGGTCGAAACGTA	23
	RT-PCR INFA-2	GGTGACAGGATTGGTCTTGTCTTTA	25
	RT-PCR INFA probe	[HEX]-TCAGGCCCCCTCAAAGCCGAG	21
Influenza B (HA gene)	RT-PCR INFB-1	AAATACGGTGGATTAAATAAAAGCAA	26
	RT-PCR INFB-2	CCAGCAATAGCTCCGAAGAAA	21
	RT-PCR INFB probe	[FAM]-CACCCATATTGGGCAATTTCCTATGGC	26

### Multiplex influenza A/B/IC RT-LAMP assay

The influenza A/B/IC multiplex RT-LAMP assay was performed with a Mmiso RNA amplification kit (Mmonitor, South Korea). The RT-LAMP reaction was prepared with 12.5 μL of 2x reaction buffer, 1.25 μL of influenza A LAMP primer mix, 0.625 μL of influenza B LAMP primer mix, 0.625 μL of internal control LAMP primer mix, 720 nM quencher solution, 2 μL of enzyme mix, and 2.5 μL of sample RNA (final reaction volume: 25 μL). The composition of the LAMP primer mix (influenza A and influenza B) included 4 μM of two outer primers (F3 and B3), 32 μM of two inner primers (FIP and BIP), 10 μM of loopB primer, 4 μM of loopF primer, and 6 μM of loopF probe primer. The composition of the internal control LAMP primer mix included 4 μM of two outer primers (F3 and B3), 32 μM of two inner primers (FIP and BIP), 10 μM of loopF primer, 4 μM of loopB primer, and 6 μM of loopB probe primer. The RT-LAMP assay was run on the CFX 96 Touch Real-Time PCR Detection System (Bio-Rad Laboratories, Hercules, CA, USA) at 60°C for 60 min. In LAMP assay, negative control (human serum RNA and distilled water) were used for setting baseline. Positive signal was determined by checking whether signal is steep or gradual considering the baseline, because baseline of LAMP assay is not stable compared to qPCR or RT-PCR. The FAM, HEX and Texas red fluorescence channels was used for detecting Influenza A, influenza B and internal control (actin beta), respectively.

### Real-time RT-PCR

To evaluate the performance of the multiplex influenza A/B/IC RT-LAMP assay, two real-time RT-PCRs, the commercial RealStar® Influenza RT-PCR Kit 2.0 (Altona Diagnostics, Hamburg, Germany) and the World Health Organization (WHO) influenza A/B primer set [33, 34] with the DiaStar OneStep Multiplex qRT-PCR Kit (SolGent Co., Ltd., Daejeon, Korea), were performed using the CFX96 Touch Real-Time PCR Detection System (Bio-Rad Laboratories). The PCR cycling conditions of the WHO influenza A/B primer set were as follows: reverse transcription at 50°C for 20 min, inactivation at 95°C for 15 min, 39 cycles of denaturation at 95°C for 30 s, and annealing with fluorescence detection at 60°C for 40 s. For the RealStar® Influenza RT-PCR Kit 2.0, the thermocycling parameters were as follows: reverse transcription at 55°C for 20 min, inactivation at 95°C for 2 min, 44 cycles of denaturation at 95°C for 15 s, annealing with fluorescence detection at 55°C for 45 s, and extension at 72°C for 15 s.

### Limit of Detection (LOD) tests of the multiplex influenza A/B/IC RT-LAMP assay

pTOP Blunt V2 plasmids, including the segment 7 partial sequences of Influenza A, were used to test the LOD of the influenza A LAMP assay. For the LOD of the influenza B LAMP assay, pTOP Blunt V2 plasmids, including the nucleoprotein gene partial sequences of influenza B, were used. pTOP Blunt V2 plasmids, including the beta actin partial sequences of human, were used to test the LOD of the Internal control LAMP assay. All plasmids were constructed by Macrogen, Inc. The plasmids were serially diluted 10-fold from 1.0 × 10^8^ copies/μL to 1.0 × 10^1^ copies/μL to determine the LOD of the multiplex influenza A/B/IC RT-LAMP assay. In addition, the LOD of the multiplex influenza A/B/IC LAMP analysis was tested using clinical samples of influenza A/H1, A/H3, and B. Clinical specimens were diluted 10 times (as much as 10^−1^ to 10^−6^ times) based on the original samples. The LOD of the multiplex influenza A/B/IC RT-LAMP analysis for clinical influenza samples was compared to those of the WHO influenza A/B primer set and RealStar® Influenza RT-PCR Kit 2.0.

## Results

### Optimization of the multiplex influenza A/B/IC LAMP primer set

Before optimization of multiplex LAMP assay, each Influenza A, B and internal control (IC, actin beta) LAMP primer set were tested with clinical samples (Influenza A H1, H1N1, H3N2, Influenza B, human serum RNA and distilled water). All three LAMP primer sets (Influenza A, B and IC) showed the no cross-reactivity (S1 Fig).

**Fig 1:**
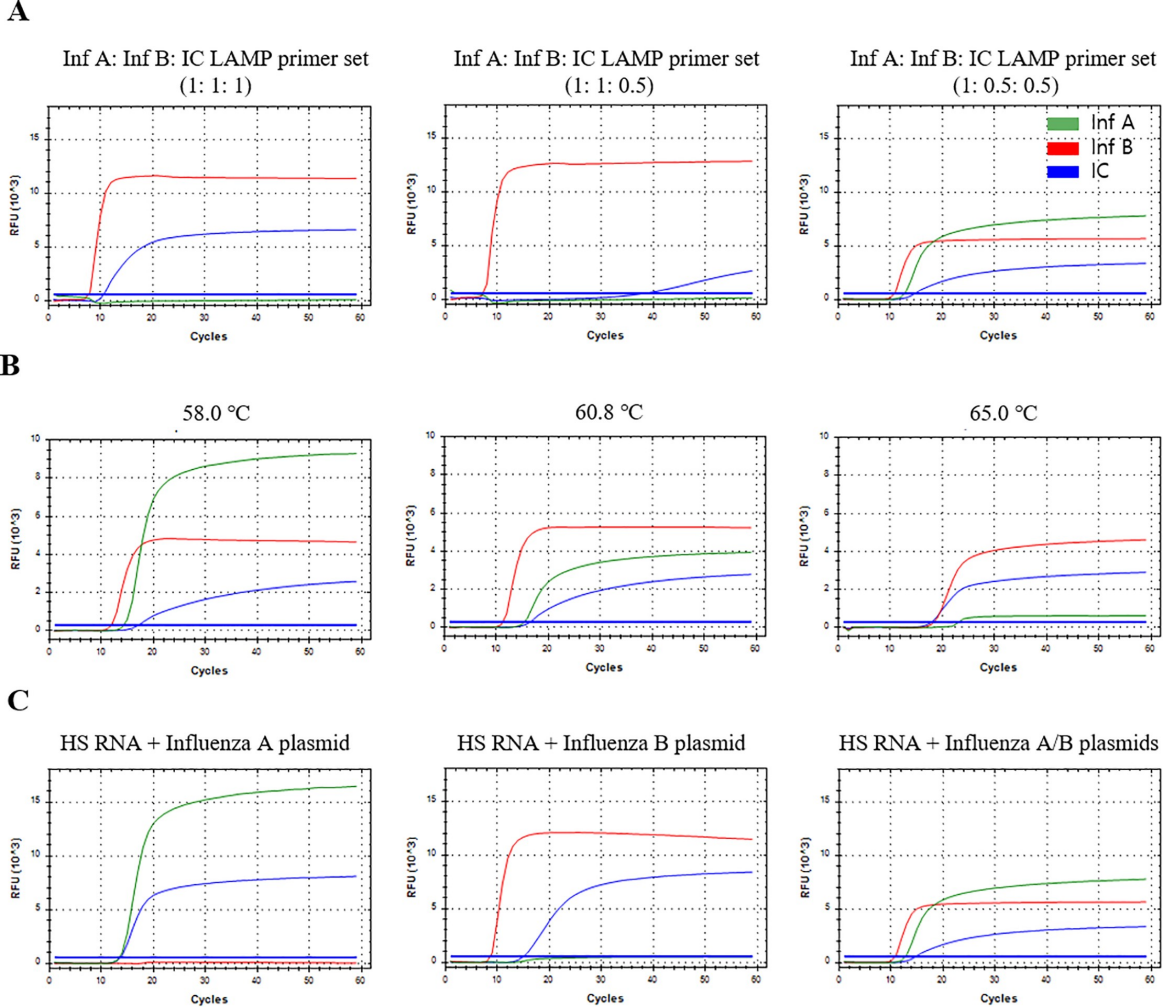
Optimization of the multiplex influenza A/B LAMP primer set. (A) Different concentration ratios of the influenza A, influenza B, and internal control primer sets (1:1:1, 1:1:0.5, and 1:0.5:0.5, respectively) for human serum RNA (HS RNA) samples spiked with influenza A and B plasmids. (B) Temperature gradient tests (58–65°C) of the multiplex influenza A/B/IC LAMP assay. (C) Performance of the multiplex influenza A/B/IC LAMP assay against HS RNA spiked with influenza A plasmid, HS RNA spiked with influenza B plasmid, and HS RNA spiked with influenza A/B plasmid (1:1).

For optimization of the multiplex influenza A/B/IC LAMP primer set, different concentration ratios of the influenza A, influenza B, and internal control primer sets (1:1:1, 1:1:0.5, and 1:0.5:0.5, respectively) were tested using human serum RNA samples spiked with influenza A and B plasmids (10^7^ copy, ratio of 1:1) (Fig 1A). As a result, three signals (influenza A, influenza B, and internal control) were detected in the ratios of 1:0.5:0.5, whereas two signals (influenza B and internal control) were detected in the ratio of 1:1:1 and 1:1:0.5. Among three ratio of LAMP primer set, the ratio of 1:0.5:0.5 showed the lowest Ct values of influenza A and influenza B were 12.58 and 10.41, respectively. Therefore, the ratios of influenza A, influenza B, and internal control LAMP primer set (1:0.5:0.5) was determined as optimum ratio for multiplex influenza A/B/IC LAMP assay. Next, temperature gradient tests (58–65°C) were performed to determine the optimal temperature of the multiplex influenza A/B/IC LAMP assay (Fig 1B). Among 8 temperatures (65, 64.6, 62.5, 60.8, 59.4, 58.5 and 58.0°C), the LAMP assay showed lowest Ct values and RFU of all fluorescence channels at 60.8°C (Ct/RFU, influenza A: 15.13/3919, influenza B: 11.02/5237 and internal control: 16.51/2745) and 59.4°C (Ct/RFU, influenza A: 14.36/6996, influenza B: 11.08/5053 and internal control: 16.73/2707). Therefore, we decided the optimal temperature for this assay to be 60°C between 60.8°C and 59.4°C. Fig 1C showed the performance of the multiplex influenza A/B/IC LAMP assay against influenza A RNA samples, influenza B RNA samples, and influenza A/B RNA mixture samples at the optimum conditions (ratio of the influenza A, influenza B, and internal control primer sets was 1:0.5:0.5 at 60°C).

### LOD tests of the multiplex influenza A/B/IC multiplex LAMP assay

**Fig 2:**
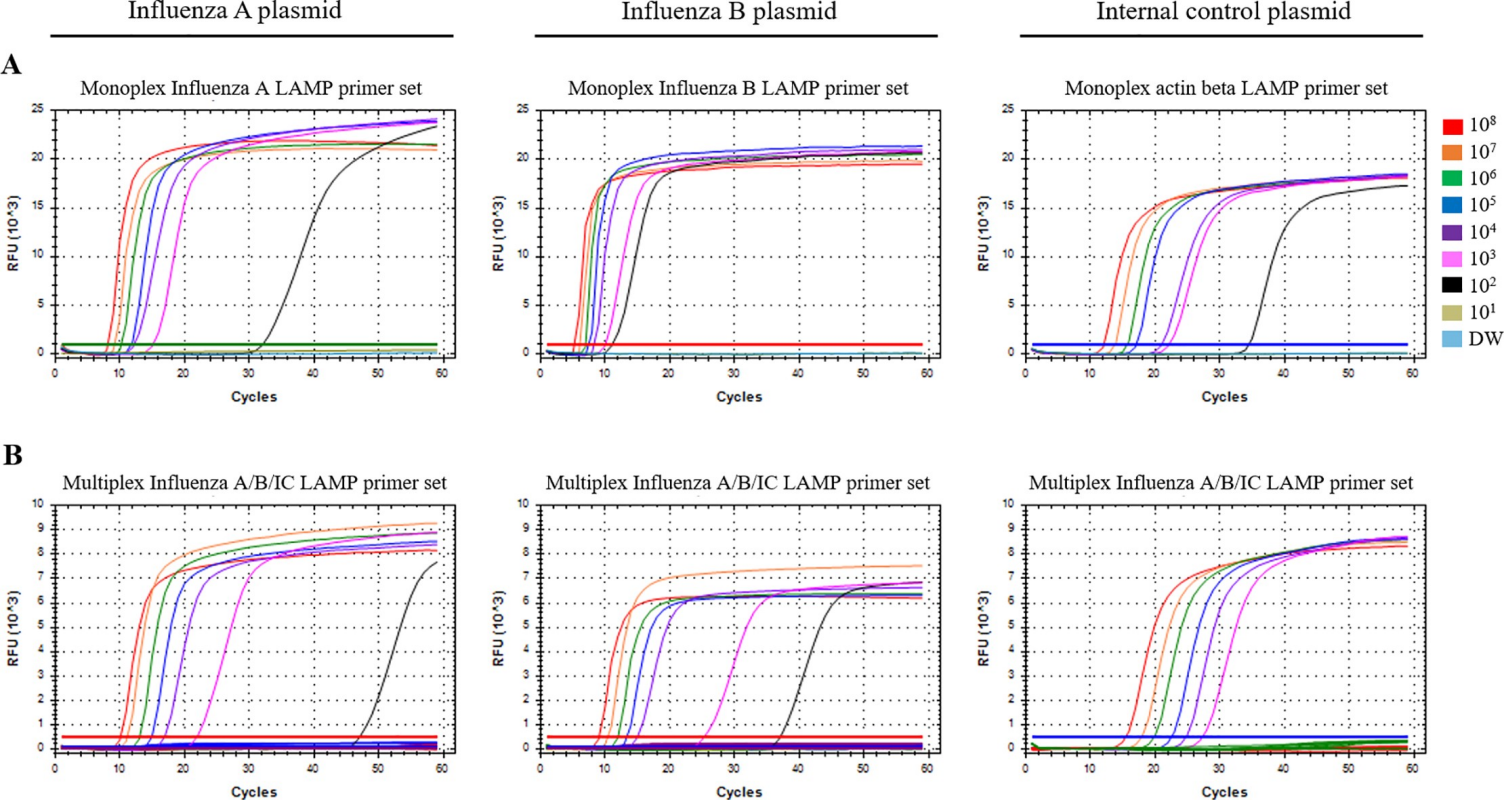
Limit of Detection (LOD) test for the monoplex and multiplex influenza A/B/IC LAMP primer set. (A) The monoplex influenza A, influenza B and IC LAMP primer sets (left, middle and right panel, respectively). (B) Multiplex influenza A/B/IC LAMP primer set (left, middle and right panel, respectively). The monoplex and multiplex influenza LAMP assays were tested with synthetic influenza A, B or beta actin plasmids ranging from 10^8^ to 10^1^ RNA copies/μL. Colors (red, orange, green, blue, purple, pink, black, olive, sky blue) indicate plasmid copy numbers/μL (1.0 × 10^8^ to 1.0 × 10^1^ copies/μL) and DW (negative control). The LOD test was repeated three times.

The analytical sensitivity of the multiplex influenza A/B/IC RT-LAMP assay was compared with monoplex LAMP primer sets [In A, In B and Internal control (IC, actin beta)] by testing synthetic RNA plasmids ranging from 10^8^ to 10^1^ RNA copies/μL (Fig 2, Table 2). In monoplex In A, In B and IC LAMP test showed all the detection limits of 1 × 10^2^ copies/μL. In multiplex influenza A/B/IC RT-LAMP assay, both influenza A and B plasmids were detected up to 1 × 10^2^ copies/μL and actin beta plasmid was detected up to 1 × 10^3^ copies/μL. As a result, multiplex influenza A/B/IC showed comparable detection limits with monoplex LAMP assay, although detection limit of IC in multiplex was higher than that of IC monoplex LAMP. Furthermore, the LOD of the RT-LAMP assay was compared with the WHO RT-PCR primer set and commercial RealStar® Influenza RT-PCR Kit 2.0 using serially diluted clinical influenza A/H1, A/H3, and B samples (range of 10^−1^ to 10^−6^; Table 3). As a result, the LOD of both the WHO RT-PCR primer set and RealStar® Influenza RT-PCR Kit 2.0 for influenza A/H1 was 10^−3^ while that of the multiplex influenza A/B/IC RT-LAMP assay was 10^−2^. For influenza A/H3, the RealStar® Influenza RT-PCR Kit 2.0 assay showed the highest sensitivity (10^−5^), and the LOD of the other two assays was 10^−4^. For influenza B, the RealStar® Influenza RT-PCR Kit 2.0 assay showed the highest sensitivity (10^−4^), and the LOD of the other two assays was 10^−3^. Overall, the detection limits tested with clinical sample dilutions were the lowest in the analysis of the RealStar® Influenza RT-PCR Kit 2.0, followed by the WHO RT-PCR primer set, and finally the multiplex influenza A/B/IC RT-LAMP assay.

**Table 2:** Limit of Detection (LOD) test for the monoplex and multiplex influenza A/B/IC LAMP primer set.

		**Monoplex RT-LAMP**		**Multiplex influenza A/B/IC RT-LAMP**					
	**Plasmid**	**Influenza A**		**Influenza A**		**Internal control**		**Influenza B**	
		**Ct**	**RFU**	**Ct**	**RFU**	**Ct**	**RFU**	**Ct**	**RFU**
Influenza A plasmid dilution sample (copies/ul)	10^8^	**9.48**	**21710**	**11.28**	**19116**	Neg	119	Neg	36
	10^7^	**10.83**	**20898**	**12.74**	**19731**	Neg	120	Neg	75
	10^6^	**12.28**	**21106**	**14.26**	**19653**	Neg	127	Neg	41
	10^5^	**13.82**	**22023**	**15.61**	**19871**	Neg	132	Neg	43
	10^4^	**15.39**	**21662**	**18.46**	**19936**	Neg	146	Neg	114
	10^3^	**17.04**	**21929**	**20.95**	**20090**	Neg	115	Neg	127
	10^2^	**33.14**	**7877**	**36.76**	**10511**	Neg	131	Neg	53
	10^1^	Neg	239	Neg	-5	Neg	3	Neg	46
	**Plasmid**	**Influenza B**		**Influenza A**		**Internal control**		**Influenza B**	
		**Ct**	**RFU**	**Ct**	**RFU**	**Ct**	**RFU**	**Ct**	**RFU**
Influenza B plasmid dilution sample (copies/ul)	10^8^	**5.35**	**20471**	Neg	651	Neg	38	**8.80**	**10283**
	10^7^	**6.17**	**20994**	Neg	480	Neg	2	**10.07**	**10588**
	10^6^	**7.07**	**21366**	Neg	400	Neg	44	**11.46**	**10394**
	10^5^	**7.85**	**22074**	Neg	301	Neg	6	**12.84**	**10526**
	10^4^	**8.56**	**21507**	Neg	279	Neg	6	**13.95**	**10641**
	10^3^	**10.27**	**21393**	Neg	256	Neg	11	**19.48**	**10601**
	10^2^	**11.17**	**6952**	Neg	182	Neg	22	**27.61**	**6528**
	10^1^	Neg	42	Neg	13	Neg	12	Neg	35
	**Plasmid**	**Internal control**		**Influenza A**		**Internal control**		**Influenza B**	
		**Ct**	**RFU**	**Ct**	**RFU**	**Ct**	**RFU**	**Ct**	**RFU**
Internal control plasmid dilution sample (copies/ul)	10^8^	**12.87**	**16074**	Neg	379	**14.98**	**7609**	Neg	354
	10^7^	**14.43**	**15591**	Neg	436	**17.41**	**7664**	Neg	151
	10^6^	**16.05**	**16109**	Neg	473	**20.26**	**8034**	Neg	33
	10^5^	**17.73**	**16123**	Neg	203	**22.27**	**7818**	Neg	56
	10^4^	**20.62**	**15877**	Neg	207	**24.95**	**7494**	Neg	53
	10^3^	**24.46**	**15653**	Neg	99	**35.10**	**5064**	Neg	-33
	10^2^	**39.34**	**9771**	Neg	13	Neg	67	Neg	-4
	10^1^	Neg	52	Neg	70	Neg	115	Neg	8

Each mean values of Ct and RFU are the average of three LAMP assay repetitions.

**Table 3:** Limit of Detection (LOD) tests of the multiplex influenza A/B/IC LAMP assay, World Health Organization (WHO) Influenza RT-PCR, and RealStar® Influenza RT-PCR Kit 2.0 for the clinical influenza A/B samples.

Subtype	RT-LAMP assay						WHO Influenza RT-PCR						RealStar® Influenza RT-PCR 2.0 (Altona)					
	RNA copies (10^n^) per reaction																	
	-1	-2	-3	-4	-5	-6	-1	-2	-3	-4	-5	-6	-1	-2	-3	-4	-5	-6
Influenza A (H1)																		
Influenza A (H3)																		
Influenza B																		

### Comparison of clinical performance between the multiplex influenza A/B/IC RT-LAMP assay, WHO RT-PCR primer set, and RealStar® Influenza RT-PCR Kit 2.0 using clinical samples

To confirm the clinical performance of the multiplex influenza A/B/IC RT-LAMP assay, the sensitivities and specificities of the assay were compared with those of the WHO RT-PCR primer set and RealStar® Influenza RT-PCR Kit 2.0 for 93 influenza A, 80 influenza B, and 100 healthy patient NP specimens (Table 4). For the influenza A H1 clinical samples (n = 11), the sensitivities of the WHO RT-qPCR primer set and RealStar® Influenza RT-PCR Kit 2.0 were 100% and 81.81%, respectively. The sensitivities of the multiplex influenza A/B/IC RT-LAMP assay were 90.90% in the influenza A channel (FAM) and 81.81% in the internal control channel. The specificities of all three assays for influenza A/H1 clinical samples were 100%. For influenza A/H3 clinical samples (n = 82), the sensitivities of the WHO RT-PCR primer set and RealStar® Influenza RT-PCR Kit 2.0 were 97.59% and 89.02%, respectively. The sensitivities of the multiplex influenza A/B/IC RT-LAMP assay were 95.12% in the influenza A channel (FAM) and 36.58% in the internal control channel. The specificities of the WHO RT-PCR primer set and multiplex influenza A/B/IC RT-LAMP assay for influenza A/H3 clinical samples were 100% while that of the RealStar® Influenza RT-PCR Kit 2.0 was 95.12%. Overall, the WHO RT-PCR primer set was found to have the highest sensitivity (97.84%) for all influenza A clinical samples (n = 93), followed by the multiplex influenza A/B/IC RT-LAMP assay (94.62%), and finally the RealStar® Influenza RT-PCR Kit 2.0 (88.17%). The specificities of the former two assays for influenza A clinical samples were 100% while that of the latter was 95.69%. In addition, the multiplex influenza A/B/IC RT-LAMP assay showed 100% of sensitivities and specificities for H5 (n = 10), H7 (n = 10) and H9 (n = 10) subtypes of avian influenza viruses (S1 Table). For influenza B clinical samples (n = 80), the multiplex influenza A/B/IC RT-LAMP assay and WHO RT-PCR primer set showed the highest sensitivities (97.50% and 97.50%, respectively) while that of the RealStar® Influenza RT-PCR Kit 2.0 was 86.25% (Table 4). The internal control channel of the multiplex influenza A/B/IC RT-LAMP assay showed 97.50% sensitivity for influenza B clinical samples. The specificities of all three assays against influenza B clinical samples were 100%. The specificities of the multiplex influenza A/B/IC RT-LAMP assay and WHO RT-PCR primer set for healthy clinical samples (non-infection; n = 100) were 100%, whereas that of the RealStar® Influenza RT-PCR Kit 2.0 was 99% (Table 4). The sensitivity of the internal control channel of the multiplex influenza A/B/IC RT-LAMP assay for healthy clinical samples was 100%.

**Table 4:** Comparison of clinical performance between the multiplex influenza A/B/IC RT-LAMP assay, WHO Influenza RT-PCR, and RealStar® Influenza RT-PCR Kit 2.0 for influenza A subtypes (H1N1 and H3N2) and influenza B viruses in the clinical samples.

Clinical samples		RT-LAMP assay			WHO Influenza RT-PCR		RealStar® Influenza RT-PCR (Altona)	
		In A (FAM)	IC (Hex)	In B (Tex)	In A (Hex)	In B (FAM)	In A (FAM)	In B (Cy5)
Inf A/H1N1 (n = 11)	P/N	10/1	9/2	0/11	11/0	0/11	9/2	0/11
	Sensitivity	**90.90%**	**81.81%**	**-**	**100%**	**-**	**81.81%**	
	Specificity	**-**	**-**	**100%**	**-**	**100%**		**100%**
Inf A/H3N2 (n = 82)	P/N	78/4	30/52	0/82	80/2	0/82	73/9	4/78
	Sensitivity	**95.12%**	**36.58%**	-	**97.59%**	-	**89.02%**	
	Specificity	-	-	**100%**	**-**	**100%**	**-**	**95.12%**
Inf A (n = 93)	P/N	88/5	39/54	0/93	91/2	0/93	82/11	4/89
	Sensitivity	**94.62%**	**41.93%**	-	**97.84%**	-	**88.17%**	
	Specificity	-	-	**100%**	**-**	**100%**	**-**	**95.69**
Inf B (n = 80)	P/N	0/80	78/2	78/2	0/80	78/2	0/80	69/11
	Sensitivity	**-**	**97.50%**	**97.50%**	**-**	**97.50%**	**-**	**86.25%**
	Specificity	**100%**	**-**	**-**	**100%**	**-**	**100%**	
Non-infection (n = 100)	P/N	0/100	100/0	0/100	0/100	0/100	1/99	0/100
	Sensitivity	**-**	**100%**	**-**	**-**	**-**		
	Specificity	**100%**	**-**	**100%**	**100%**	**100%**	**99%**	**100%**

The sensitivities and specificities were calculated by taking the Anyplex™ II RV16 Detection as is standard. P/N: positive/negative ratio.

### Cross-reactivity test

To confirm the possibility of cross-reactivity with other infectious viruses, 41 respiratory virus samples, including 4 RSV A, 4 RSV B, 4 adenovirus, 4 PIV(1–4), 9 coronavirus (OC43/HKU1, NL63, and 229E), 4 HBoV, 4 HEV, 4 HRV, and 4 MPV samples, were tested using the multiplex influenza A/B/IC RT-LAMP assay, WHO RT-PCR primer set, and RealStar® Influenza RT-PCR Kit 2.0 (Table 5) [33, 34]. All three molecular diagnostic tests showed no cross-reactivity with other infectious viruses, suggesting that these tests can specifically detect influenza viruses.

**Table 5:** Cross-reactivity of the multiplex influenza A/B/IC RT-LAMP assay against other human infectious viruses.

Virus	No	RT-LAMP			qRT-PCR (WHO)		qRT-PCR (RealStar®)	
		In A (FAM)	IC (Hex)	In B (Tex)	In A (Hex)	In B (FAM)	In A (FAM)	In B (Cy5)
HEV	4	0/4	4/4	0/4	0/4	0/4	0/4	0/4
AdV	4	0/4	3/4	0/4	0/4	0/4	0/4	0/4
PIV (1–4)	4	0/4	4/4	0/4	0/4	0/4	0/4	0/4
MPV	4	0/4	4/4	0/4	0/4	0/4	0/4	0/4
HboV	4	0/4	2/4	0/4	0/4	0/4	0/4	0/4
HRV	4	0/4	3/3	0/4	0/4	0/4	0/4	0/4
229E	3	0/3	3/3	0/3	0/3	0/3	0/3	0/3
NL63	3	0/3	3/3	0/3	0/3	0/3	0/3	0/3
OC43	3	0/3	3/3	0/3	0/3	0/3	0/3	0/3
RSV A	4	0/4	4/4	0/4	0/4	0/4	0/4	0/4
RSV B	4	0/4	4/4	0/4	0/4	0/4	0/4	0/4

HEV: human enterovirus, AdV: adenovirus, PIV(1–4): parainfluenza virus(1–4), MPV: human metapneumovirus, HBoV: human bocavirus, HRV: human rhinovirus, 229E: human coronavirus 229E, NL63: human coronavirus NL63, OC43: human coronavirus OC43, RSV A: respiratory syncytial virus A, RSV B: respiratory syncytial virus B.

## Discussion

Since many respiratory viruses, including influenza viruses, can cause similar symptoms, it is difficult for clinicians to distinguish one virus from another [35]. Given the annual morbidity and mortality caused by influenza viruses, there is an urgent need for sensitive and convenient laboratory methods to identify influenza virus subtypes in clinical care and infection control [36, 37]. There are immunodiagnostic kits that can be utilized quickly, but their sensitivity and specificity are too poor; thus, PCR methods are currently used to make accurate diagnoses [38]. However, PCR-based target gene detection requires bulky and expensive equipment as well as highly skilled technicians [39]. Several isothermal amplification methods, such as HDA, RPA, and LAMP, have been developed for on-site diagnosis of infectious pathogens [40–42]. Among them, LAMP is a promising method that has been utilized to detect a variety of pathogens [43, 44]. Recently, a variety of multiplex RT-LAMP methods have been developed to detect influenza viruses by using annealing temperature, nanoparticle hybridization, one-pot colorimetric visualization and immunochromatographic strip etc [45–48]. However, their multiplex system amplified each type of influenza individually or consist of two steps, which are lamp assay and rapid test. Thus, it may take a more time when diagnosis a large number of clinical samples. Thus, it can take a more time to diagnose a large number of clinical samples. In addition, it is known that LAMP assay is easy to contaminate [49, 50]. In this study, we developed the one tube-multiplex influenza A/B/IC LAMP assay, including an internal control (actin), for the detection of influenza A/H1, A/H3, and B using newly designed assimilating probes, which has advantages for reducing test time and risk of contamination.

Our results showed that the multiplex influenza A/B/IC RT-LAMP assay had 100% analytical specificity for the identification of influenza A/H1, A/H3, and B viruses, and there was no cross-reaction with other genetically or clinically related control viruses tested in this study. The multiplex influenza A/B/IC RT-LAMP assay for influenza A clinical samples (n = 93) had a sensitivity and specificity of 94.62% and 100%, respectively. However, the multiplex influenza A/B/IC RT-LAMP assay showed a 90% sensitivity for influenza A/H1 because out of the 11 specimens that were positive for influenza A/H1, only 10 were determined by the assay to be positive. Further testing with additional clinical specimens is needed to evaluate the clinical performance characteristics of the multiplex influenza A/B/IC RT-LAMP assay to address the issue of the small sample size used (n = 10). In addition, the internal control signal showed in a very low sensitivity for influenza A clinical samples but not influenza B samples and negative clinical samples. This result might be that LAMP amplification reagents were consumed for the amplification of influenza A or influenza A amplification products interrupt the amplification of the internal control. Interestingly, the multiplex influenza A/B/IC RT-LAMP assay had a lower detection sensitivity for diluted clinical samples than the RealStar® Influenza RT-PCR Kit 2.0 but higher sensitivity for original clinical samples. These results suggest that the multiplex influenza A/B/IC RT-LAMP assay had a higher sensitivity for various influenza genetic sequences while the RealStar® Influenza RT-PCR Kit 2.0 had a higher sensitivity for specific influenza genetic sequences.

RT-LAMP analysis is one of the most promising diagnostic tools for use in the field since it does not require sophisticated and expensive equipment or skilled personnel [51]. The multiplex method developed in this study can diagnose influenza A and B within 60 min using multiple fluorescence. In order to utilize the multiplex influenza A/B/IC RT-LAMP assay in the field, it is necessary to use an isothermal amplification device that detects portable multiple fluorescence. Most of the field isothermal amplifiers currently available have been developed as single channel. However, two-channel isothermal amplifiers (Genie III; OptiGene, West Sussex, UK) have recently been developed and marketed by Chayon Laboratories, Inc. Since there are no field isothermal amplifiers with three channels yet, the influenza A/B and internal control LAMP kits cannot be used. However, by excluding the internal control, influenza A and B can now be diagnosed in the field using commercially available isothermal amplifiers. In addition, conventional RNA extraction methods, which extract RNA using centrifuges from NP swabs or aspirate samples collected from suspected influenza patients, are time-consuming and may be potentially contaminated. Therefore, it is expected that influenza field diagnosis can be performed more effectively by using an RNA extraction chip [52] or the magnetic bead-nucleic acid extraction method [53].

In this study, we developed a multiplex real-time RT-LAMP assay that can diagnose influenza A and influenza B with one step. The multiplex influenza A/B/IC RT-LAMP assay using the probe-quencher that compensates for the disadvantages of LAMP, such as false positive diagnoses, shows similar sensitivity and specificity to the WHO RT-PCR primer set. Since LAMP takes less time (within 60 min) than conventional RT-PCR, the multiplex influenza A/B RT-LAMP assay can be used as an efficient method in on-site molecular diagnostic kits.

## Supporting information

S1 Fig Gel electropherograms of influenza A, B and IC (actin beta) RT-LAMP products. Influenza A/B virus clinical samples, non-infection human serum RNA and distilled water (DW) were tested by RT-LAMP assays with monoplex influenza A RT-LAMP primer set (A), monoplex influenza B RT-LAMP primer set (B) and monoplex IC (actin beta) RT-LAMP primer set (C). Lane M: DNA ladder marker, Lane 1: Influenza A/H1, Lane 2: Influenza A/H1N1, Lane 3: Influenza A/H3N2, Lane 4: Influenza B, Lane 5: Non-infection human serum RNA and Lane 6: DW (negative control).
(TIF)

S1 Table Sensitivities and specificities of the multiplex influenza RT-LAMP assay for H5, H7 and H9 subtypes of avian influenza viruses. (DOCX)
