# Supplementary figures and images for: Development of a multiplex isothermal amplification molecular diagnosis method for on-site diagnosis of influenza
Source: PLoS One. 2020 Sep 11;15(9):e0238615. doi: 10.1371/journal.pone.0238615 (PMC7485819; doi:10.1371/journal.pone.0238615)

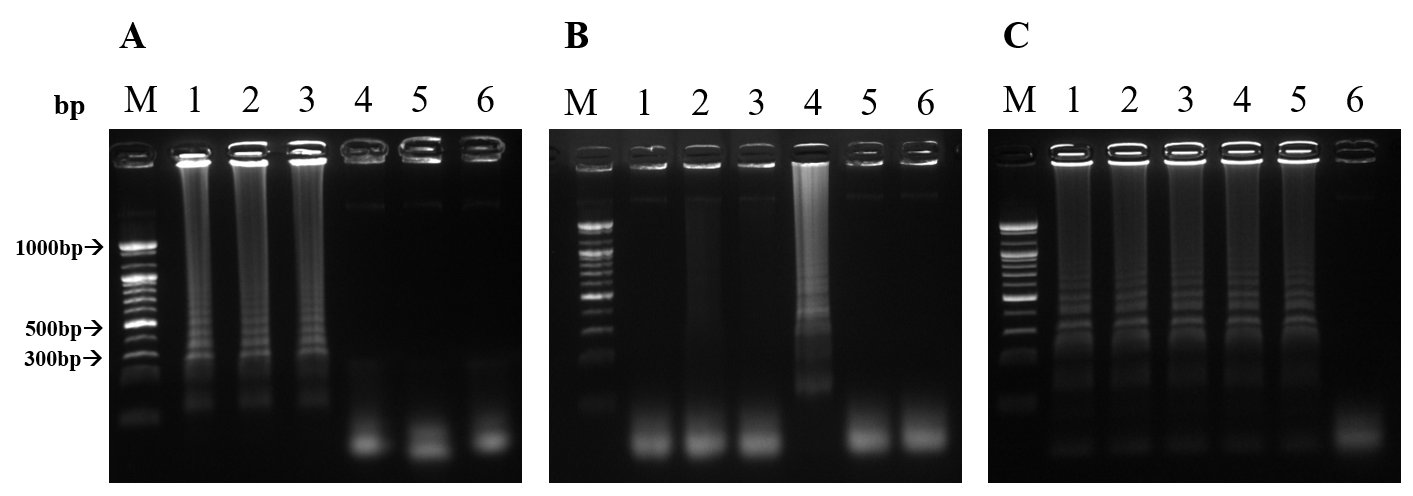

Supplement: S1 Fig — Influenza A/B virus clinical samples, non-infection human serum RNA and distilled water (DW) were tested by RT-LAMP assays with monoplex influenza A RT-LAMP primer set (A), monoplex influenza B RT-LAMP primer set (B) and monoplex IC (actin beta) RT-LAMP primer set (C). Lane M: DNA ladder marker, Lane 1: Influenza A/H1, Lane 2: Influenza A/H1N1, Lane 3: Influenza A/H3N2, Lane 4: Influenza B, Lane 5: Non-infection human serum RNA and Lane 6: DW (negative control). (TIF) [file pone.0238615.s001.tif]
