# Supplementary material for: Development of a multiplex isothermal amplification molecular diagnosis method for on-site diagnosis of influenza
Source: PLoS One. 2020 Sep 11;15(9):e0238615. doi: 10.1371/journal.pone.0238615 (PMC7485819; doi:10.1371/journal.pone.0238615)
Supplement: S1 Table — (DOCX) [file pone.0238615.s002.docx]

**S1 Table. Sensitivities and specificities of the multiplex influenza RT-LAMP assay for H5, H7 and H9 subtypes of avian influenza viruses.**

| **Avian Influenza**  **Clinical samples** |  | **RT-LAMP assay** | | |  |
| --- | --- | --- | --- | --- | --- |
|  |  | **In A (FAM)** | **IC (Hex)** | **In B (Tex)** |  |
| Inf A/H5  (n=10) | P/N | 10/0 | 0/10 | 0/10 |  |
|  | Sensitivity | **100%** | **-** | **-** |  |
|  | Specificity | **-** | **100%** | **100%** |  |
| Inf A/H7  (n=10) | P/N | 10/0 | 0/10 | 0/10 |  |
|  | Sensitivity | **100%** | **-** | **-** |  |
|  | Specificity | **-** | **100%** | **100%** |  |
| Inf A/H9  (n=10) | P/N | 10/0 | 0/10 | 0/10 |  |
|  | Sensitivity | **100%** | **-** | **-** |  |
|  | Specificity | **-** | **100%** | **100%** |  |
